# SMRT sequencing of the *Campylobacter coli* BfR-CA-9557 genome sequence reveals unique methylation motifs

**DOI:** 10.1186/s12864-015-2317-3

**Published:** 2015-12-21

**Authors:** Andreas E. Zautner, Anne-Marie Goldschmidt, Andrea Thürmer, Jörg Schuldes, Oliver Bader, Raimond Lugert, Uwe Groß, Kerstin Stingl, Gabriela Salinas, Thomas Lingner

**Affiliations:** Institute for Medical Microbiology, University Medical Center Göttingen, Kreuzbergring 57, D-37075 Göttingen, Germany; Institute for Microbiology and Genetics, Department of Genomic and Applied Microbiology and Göttingen Genomics Laboratory, Georg-August University Göttingen, Grisebachstr. 8, D-37077 Göttingen, Germany; Federal Institute for Risk Assessment (BfR), Department of Biological Safety – National Reference Laboratory for Campylobacter, D-12277 Berlin, Germany; Microarray and Deep-Sequencing Core Facility, University Medical Center Göttingen, Justus-von-Liebig-Weg 11, D-37077 Göttingen, Germany

**Keywords:** *Campylobacter coli*, Genome, Methylation, Motifs, Methylome, Restriction modification systems, Isoschizomer digestion assay, SMRT sequencing, PacBio

## Abstract

**Background:**

*Campylobacter species* are the most prevalent bacterial pathogen causing acute enteritis worldwide. In contrast to *Campylobacter jejuni,* about 5 % of *Campylobacter coli* strains exhibit susceptibility to restriction endonuclease digestion by *Dpn*I cutting specifically 5’-G^m^ATC-3’ motifs. This indicates significant differences in DNA methylation between both microbial species.

The goal of the study was to analyze the methylome of a *C. coli* strain susceptible to *Dpn*I digestion, to identify its methylation motifs and restriction modification systems (RM-systems), and compare them to related organisms like *C. jejuni* and *Helicobacter pylori*.

**Results:**

Using one SMRT cell and the PacBio RS sequencing technology followed by PacBio Modification and Motif Analysis the complete genome of the *Dpn*I susceptible strain *C. coli* BfR-CA-9557 was sequenced to 500-fold coverage and assembled into a single contig of 1.7 Mbp. The genome contains a CJIE1-like element prophage and is phylogenetically closer to *C. coli* clade 1 isolates than clade 3. 45,881 6-methylated adenines (ca. 2.7 % of genome positions) that are predominantly arranged in eight different methylation motifs and 1,788 4-methylated cytosines (ca. 0.1 %) have been detected. Only two of these motifs correspond to known restriction modification motifs. Characteristic for this methylome was the very high fraction of methylation of motifs with mostly above 99 %.

**Conclusions:**

Only five dominant methylation motifs have been identified in *C. jejuni*, which have been associated with known RM-systems. *C. coli* BFR-CA-9557 shares one (RAATTY) of these, but four ORFs could be assigned to putative Type I RM-systems, seven ORFs to Type II RM-systems and three ORFs to Type IV RM-systems. In accordance with *Dpn*I prescreening RM-system IIP, methylation of GATC motifs was detected in *C. coli* BfR-CA-9557. A homologous IIP RM-system has been described for *H. pylori*. The remaining methylation motifs are specific for *C. coli* BfR-CA-9557 and have been neither detected in *C. jejuni* nor in *H. pylori*.

The results of this study give us new insights into epigenetics of Campylobacteraceae and provide the groundwork to resolve the function of RM-systems in *C. coli*.

**Electronic supplementary material:**

The online version of this article (doi:10.1186/s12864-015-2317-3) contains supplementary material, which is available to authorized users.

## Background

*Campylobacteriosis* is the most prevalent form of bacterial acute enteritis worldwide. In symptomatic cases it is characterized by a prodromal phase with fever, vomiting, and headaches followed by watery or bloody diarrhea and abdominal cramps [1, 2]. In consequence of acute enteritis, extraintestinal post-infectious sequelae, namely, the Guillain-Barré syndrome, inflammatory bowel disease, and reactive arthritis may occur [3, 4]. The average incidence reported in the European Union was 64.8 per 100,000 population in 2013 [5], in the USA 14.3 cases per 100,000 population in 2012, and in China 161 cases per 100,000 population in urban areas compared to 37 cases per 100,000 population in rural areas [6]. In Europe, 80.6 % were reported to have been caused by *Campylobacter jejuni* and 7.1 % by *Campylobacter coli* [5]*.*

*C. coli* is phylogenetically subdivided into three clades [7, 8]: clade 1 isolates commonly colonize swine but can also be isolated from poultry and humans, although less frequently. Clades 2 and 3 are typically isolated from environmental waters [8, 9].

At the moment, seven completed *C. coli* chromosomal genome sequences [10–13], several scaffold genomes, and various contigs have been deposited in the NCBI Genome database [14–17]. The completed genome sequences, range from 1.685 to 1.872 Mb, have a G + C content of about 31 to 32 %, and contain 1715 – 1970 predicted genes including 1642 – 1861 protein coding ORFs [10–13].

One of the major epigenetic mechanisms in prokaryotes is DNA methylation [18]. DNA methylation patterns influence gene expression [19], through silencing of transcription [20, 21] as well as DNA replication initiation [22, 23] and mismatch repair [24]. DNA methylation also serves as a protection of the host genome against extraneous DNA [18] through restriction-modification systems (RM-systems). RM-systems consist of two components: (i) a restriction endonuclease that recognizes a specific DNA motif and (ii) a cognate DNA methyltransferase that methylates the same DNA, preventing its cleavage by the restriction endonuclease [25]. The majority of RM-systems can be categorized into four types [25–29]:

Type I RM-systems typically consist of three types of subunits: two restriction endonuclease subunits (R), which facilitate DNA cleavage, one specificity subunit (S) for recognition of specific DNA sequence motifs, and two DNA methylase subunits (M) that catalyse N^6^ adenine methylation [30, 31]. This composition enables Type I RM-systems to digest unmethylated DNA, whereas hemimethylated DNA is further methylated and fully methylated DNA is insusceptible to restriction [32].

Type II RM-systems are mostly composed of two homodimeric R subunits and a separated M subunit. The R and M subunits recognize the same DNA motif, which is typically a 4–8 bp palindrome [33].

Type III RM-systems are comprised of two modification (Mod) subunits and two restriction (R) subunits. Type III RM-systems must bind to two inversely oriented copies of its 5–6 bp asymmetric recognition motif. Cleavage of unmethylated DNA typically occurs 25–27 bp away from the binding sites [34].

Type IV RM-systems consist of two separate R subunits cleaving DNA that contains methylated, hydroxymethylated or glucosyl-hydroxymethylated cytosines. Cleavage typically occurs 30 bp away from one of the binding sites [35]. Furthermore, there exist many orphan DNA methylases that are not part of a RM-system e.g. DNA adenine methylase (Dam) and cell cycle-regulated DNA methyltransferase (CcrM) [29].

It must be considered that the genes encoding for particular components of a RM-system are diverse within microbial species i.e. *C. jejuni* and *C. coli* [36–38]. In *C. jejuni* the putative Type I RM-system locus (genes *cj1549*–*cj1553; hsd* locus) reveals significant diversity regarding gene order, chromosomal location, intervening ORFs and gene sequence leading to the classification of the *C. jejuni hsd* loci into at least three families, namely the IAB, IC, and IF family. Especially sequence variations in the *hsdS* gene suggest at least 30 different target sequence specificities and therewith differences in DNA methylation [39]. Holt and coworkers demonstrated that *cj1051c* (*cjeI*) encodes an active restriction-modification Type IIG enzyme in *C. jejuni* that significantly decreases transformation efficiency with plasmids [40]. Additionally it was shown that Type IIS restriction modification enzyme Cj0030/Cj0031 is subject to phase variable gene expression due to mutations in polyC/G tracts [41–43]. Additionally *cj0139*/*cj0140* encode a putative 5-methylcytosine-restriction system, *cj0690c* a Type II RM-system and *cj0979c* a nuclease that could be part of a RM-system [41, 42].

Furthermore it was shown for *C. jejuni* that gene product of *cj1461* is a N^6^-adenine-specific DNA methyltransferase that is not a Dam homologue and not part of a RM-system. Knockout of *cj1461* affects flagellar appearance, motility, adherence, and invasion indicating its role for epigenetic control of proteins involved in these processes [44]. Further non-ubiquitous modification/methyltransferase gene loci have been observed in *C. jejuni* ST-677 isolates, namely *fixL* (*cjj5070_14950c*) that showed homology with DNA adenine/modification methylases in *Campylobacter rectus*, a homolog of *iceA1*/*nlaIII* (*cjj5070_14940c*), *cjj5070_14910c* predicted as ulcer associated adenine-specific DNA methyltransferase, which is an ortholog of the CATG-specific methyltransferase *hpyIM* of *H. pylori*, and the orphan DNA methyltransferase in ORF *cjj5070_08940* [45]. Three strain-specific RM-systems have been reported for *C. jejuni* ST403 complex: R. HinPI restriction endonuclease (*cje135_03870*), Modification methylase Hhal (*cje135_03865*), and R.Pab1 restriction endonuclease (*cje135_02348*) [46].

In contrast to *C. coli,* Type III RM-systems have been reported for *C. jejuni* subsp. *jejuni*, *C. jejuni* subsp. *doylei*, *Campylobacter lari*, and *Campylobacter upsaliensis* [47].

Until to date the methylomes of *C. jejuni* subsp. *jejuni* (3 isolates) and of the related microbial species *Helicobacter pylori* (2 isolates) have been analyzed [48–50], but so far no *C. coli* methylome has been examined.

Studies performing isoschizomer digestion assays indicated differences in methylation at GATC sites in genomic DNA of *C. coli* isolates, which suggested host-associated DNA modification systems [51, 52].

In this study we analyzed the first methylome of *C. coli* using SMRT DNA sequencing. This approach facilitates analysis of methylation motifs as well as RM-system gene loci in parallel. For SMRT sequencing a *C. coli* isolate was selected, which was tested positive for GATC site methylation isoschizomer digestion assay screening.

## Results and discussion

### SMRT sequencing and annotation

Screening of 50 *C. coli* isolates performing 5’-G^(m)^ATC-3’-specific isoschizomer digestion assays revealed six bacterial isolates showing Dam activity. Of these six isolates, the isolate BfR-CA-9557 was selected for SMRT sequencing, since it reproducibly tested positive in five biological independent analyses. Furthermore, its origin from broiler hearts sampled at a retail store in Berlin, Germany, indicates its relevance for food hygiene and thus for the infection of humans.

MLST typing revealed sequence type ST-1589 and clonal complex CC-828. Accordingly, it is a potentially human pathogenic isolate of clade 1.

Susceptibility testing indicated a quinolone resistant phenotype (zone diameter 0 mm in disc diffusion; minimal inhibition concentration (MIC) measured by broth microdilution: 16 μg/ml ciprofloxacin, 32 μg/ml nalidixic acid). In contrast, the isolate was tested susceptible for macrolides, tetracyclines and aminoglycosides (MIC values: 2 μg/ml erythromycin, <=0,5 μg/ml tetracycline, 1 μg/ml gentamicin, 2 μg/ml streptomycin).

Sequencing of the *C. coli* isolate using one Pacific Bioscience SMRT cell resulted in 74,742 continuous long reads (CLR) with an average (total) length of 14,514 (1.08°10^9^) base pairs (Additional file 1: Figure S1). 142,135 subreads (i.e. individual fragments) of high quality and an average length of 7,602 bp could be extracted from the CLRs.

Utilizing the Pacific Bioscience high-quality *de novo* genome assembly algorithm (HGAP.2), we obtained a single polished contig of 1,720,506 base pairs with an average 500-fold coverage and a confidence score of >99.99 % (Additional file 2: Figure S2). The G + C content of the contig was 31.4 % and the most closely related genome sequence available in the NCBI genome database was that of *C. coli* 15–537360 (taxonomy ID 1358410, 87 % coverage). No plasmids were detected in this isolate.

Application of the NCBI/RAST/Prodigal annotation pipelines resulted in 1637/1769/1797 predicted coding sequences and 275/366/453 (16.8/20.7/25.2 %) predicted hypothetical proteins. (GenBank ID: CP011777, Additional files 3 and 4). Furthermore, 54 RNA genes (44 tRNA/9 rRNA/1 ncRNA) were predicted by NCBI, 52 RNAs (43 tRNA/9 rRNA) by RAST, and 54 RNAs (44 tRNA/9 rRNA/1 tmRNA) by Prodigal.

**Fig. 1:**
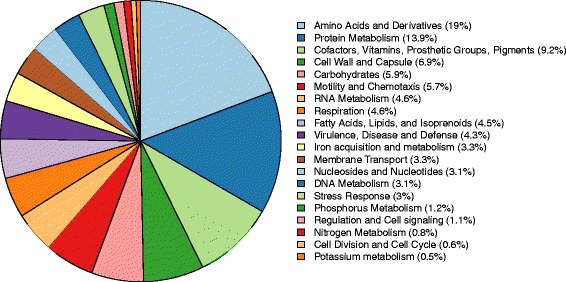
Pie chart representing RAST subsystems identified in the BFR-CA-9557 genome. The 20 most abundant subsystems on the “category” level as identified by RAST are represented by a particular color indicated at the right-hand side of the figure

The RAST subsystem coverage was 57 % (1003 genes), of which amino acid metabolism (296 of 1557 associated terms, 19.0 %), protein metabolism (216 terms, 13.9 %), and cofactors, vitamins, prosthetic groups, and pigments (144, 9.2 %) represented the largest groups (Fig. 1).

**Fig. 2:**
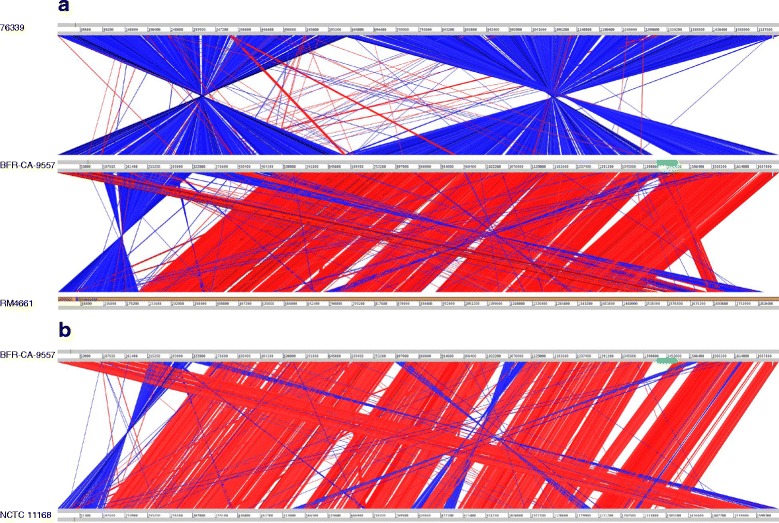
Comparison of *C. coli* BFR-CA-9557 genome to those of *C. coli* RM4661 and 76339 **a** as well as *C. jejuni* NCTC 11168 **b** using the Artemis Comparison Tool (**ACT**). Comparison of the BFR-CA-9577 genome to those of *C. coli* clade 1 strain RM4661 and *C. coli* clade 3 strain 76339 as well as to the genome of *C. jejuni* NCTC 11168 indicates that the clade 1 strain BFR-CA-9577 genome organization is more related to *C. coli* clade 1 and *C. jejuni* strains than to clade 3 strains. The CJIE1-homologue prophage in the BFR-CA-9557 genome is indicated in green

Figure 2a shows a comparison of the BfR-CA-9557 genome sequence to other *C. coli* genomes (76339, clade 3 and RM4661, clade 1) using the Artemis tool. Here, homologous regions as identified by BLAST are indicated by red (aligned in the same direction) or blue line segments (opposite direction). It can be seen that *C. coli* 76339 mainly shows three stretches of inverse homologous regions to BfR-CA-9557 and lacks similarity in the region of the identified CJIE1 (green box, see also below). RM4661 displays a long stretch of close homology with minor gaps, but also inverse homologous segments within the plasmid and the beginning and end of the chromosome. A similar pattern can be observed for the comparison of BFR-CA-9557 to *C. jejuni* NCTC 11168 (Fig. 2b). This indicates that *C. coli* BfR-CA-9557 is phylogenetically closer to clade 1 and therewith to *C. jejuni* islolates than to clade 3 *C. coli* isolates.

### Poly-G/C tracts and the capsular polysaccharid gene locus

Analysis of poly-G/C tracts within the contig of BfR-CA-9557 revealed 17 locations with homopolymeric stretches of at least eight G or C nucleotides (Tables 1 & 2). Most of these tracts occur in the vicinity of hypothetical proteins, however, two motifs could be identified within the capsular polysaccharid gene locus (cps) close to ORFs identified as glycosyltransferases (EC 2.4.99.-) by RAST. Other ORFs with neighboring poly-G/C motifs comprise e.g. transferases and ligases.

**Table 1:** Positions of poly-G tracts in the BFR-CA-9557 genome

Poly G on forward strand			Pos. rel. to		ORF location		
Position	Length	RAST annotation of closest ORF	ATG	STOP	Start	Stop	Strand
46933	9	hypothetical protein	−52	−2292	46985	49225	+
258726	10	FIG 00470070: hypothetical protein	−15	−1924	258741	260650	+
689956	10	UDP-N-acetylmuramoylalanine--D-glutamate ligase (EC 6.3.2.9)	−6	−1232	691179	689971	-
851085	10	FIG 00470965: hypothetical protein	556	−671	850529	851756	+
1562460	10	Putative transferase	−48	−590	1562508	1563050	+
1565336	9	FIG 00469667: hypothetical protein	251	−423	1565085	1565759	+
1575332	10	Motility accessory factor	167	−1780	1575165	1577112	+
1645063	9	Ferrous iron transport protein B	1576	−269	1643487	1645332	+

Poly-G/C tracts were searched in the BFR-CA-9557 genome using regular expression describing at least 8 consecutive G/C. The first two columns denote the genome location and length of the expression found. Column 3 shows the RAST annotation of the ORF closest to the homopolymeric stretch. Column 4 and 5 represent the relative position of the stretch to the ORF’s start (4) and Stop codon (5), with negative numbers representing upstream locations. Columns 6 to 8 denote the location and orientation of the ORF

**Table 2:** Positions of poly-C tracts in the BFR-CA-9557 genome

Poly C on forward strand			pos. rel. to		ORF location		
Position	Length	RAST annotation of closest ORF	ATG	STOP	Start	Stop	Strand
442261	11	Putative lipoprotein of ferric iron transporter system	139	−3	442254	442132	-
1099189	10	hypothetical protein	739	−7	1099187	1098459	-
1257487	9	Phosphoglycerol transferase	1728	−256	1257735	1255767	-
1427910	9	Filamentous haemagglutinin domain protein	1554	−9	1427911	1426364	-
1547848	9	FIG 00469527: hypothetical protein	637	−596	1548436	1547219	-
1587049	8	hypothetical protein	679	−575	1587617	1586377	-
1607917	9	FIG 00470049: hypothetical protein	2305	34	1607875	1605620	-
1660758	9	CMP-N-acetylneuraminate-beta-galactosamide-alpha-2,3-sialyltransferase (EC 2.4.99.-)	64	−620	1661370	1660702	-
1668862	8	CMP-N-acetylneuraminate-beta-galactosamide-alpha-2,3-sialyltransferase (EC 2.4.99.-)	40	−1587	1670442	1668829	-

Poly-G/C tracts were searched in the BFR-CA-9557 genome using regular expression describing at least 8 consecutive G/C. The first two columns denote the genome location and length of the expression found. Column 3 shows the RAST annotation of the ORF closest to the homopolymeric stretch. Column 4 and 5 represent the relative position of the stretch to the ORF’s start (4) and Stop codon (5), with negative numbers representing upstream locations. Columns 6 to 8 denote the location and orientation of the ORF

The cps locus of BfR-CA-9557 ranges from 1,656,271 to 1,691,672 (1,660,702-1,686,508 excluding flanking *kps* regions). Spanning 35,401 bp and encoding 29 ORFs (25,806 bp; 21 ORFs; excluding flanking kps regions) it is of comparable size to the largest known other *Campylobacter* cps cluster, which measures 38 kb excluding flanking *kps* regions (*C. jejuni* strain X) [53]. The cps loci of different *C. coli* and *C. jejuni* strains are very variable in size and gene content and there are only three genes that are comparatively conserved: the capsular polysaccharide export system periplasmic protein gene KpsD, the GDP-mannose 4,6-dehydratase gene and the capsular polysaccharide biosynthesis/export periplasmic protein *wcbA*/*kpsC*. The highest sequence similarities exist to the cps clusters of *C. coli* strains RM4661 (query coverage 71 %, identity 99 %) and FB1 (query coverage 54 %, identity 99 %). Compared to the cps locus of strain X, query coverage of 28 % (identity 87 %) was observed. The genes for *kpsC*, *hddC*, *gmhA2*, *hddA*, *dmhA*, *fcl-1*, *fcl-2*, hypothetical protein *x.25* and *kpsF* are present in both cps clusters.

### *N*-linked flagellar glycosylation locus

The *N*-linked flagellar glycosylation locus extends from 1,319,815-1,332,144 (12,329 bp). It consist of 10 ORFs and is 99 % identical to the corresponding locus in other *C. coli* genomes e.g. RM4661 (clade 1) or 76339 (clade 3). In comparison to other *C. jejuni* strains (e.g. M1 and 81116), insertion of the lipid carrier UDP-*N*-acetylgalactosaminyltransferase gene and an α-1,4-*N*-acetylgalactosamine transferase PglH gene was observed.

Directly upstream of the *N*-linked flagellar glycosylation locus the lipooligosaccharide biosynthesis locus (LOS locus) is located. The LOS locus extends from the UDP-glucose 4-epimerase gene *galE* to the D-glycero-D-manno-heptose 1,7-bisphosphate phosphatase gene *rfaD*. The LOS locus is one of the more variable regions in *Campylobacter* genomes. LOS loci containing 8 (RM2095) to 19 (81116) ORFs have been described and depending on gene content and organization 19 different LOS classes (A-S) have been defined [54, 55]. The LOS locus of BfR-CA-9557 ranges from position 1,332,138 to 1,349,328 (17,190 bp) and contains 17 ORFs. No sialyltransferase *cstII/III* homologue genes and no *N*-acetylgalactosaminyltransferase *neuABC* homologue genes are present in this cluster and therefore BfR-CA-9557 expresses an unsialylated LOS. It does not exactly belong to one of the LOS classes described for *C. jejuni* but it is closer related to the LOS classes E, P, O, and H. Remarkably, the *waaM* and *waaV* homologue genes are immediate neighbours, therefore the established sequencing approach would have been difficult due to problems resolving repetitive genomic regions [54].

### Virulence-associated genes

Neither dimethylsulfoxide (DMSO) reductase systems nor gamma glutamyltranspeptidase (*ggt*) gene could be detected in the BfR-CA-9557 genome, which is typical for a clade 1 *C. coli* isolates, but three genes homologous to the iron transport protein TonB were present in the BfR-CA-9557 genome, which has been reported as typical for clade 2 and 3 *C. coli* isolates [13]. The cytolethal distending toxin (*cdt*) operon contains all three subunits and is therewith complete.

### *Campylobacter jejuni* integrated element 1-like element & CRISPR elements

At position 1,427,993-1,467,476 we could identify a 39,483 bp sized *Campylobacter jejuni* integrated element 1 (CJIE1)-homologue prophage that shows 96 % identity (at 86 % query coverage) to CJIE1 described in *C. jejuni* RM1221 [45]. No CRISPR elements were found by any method.

### Methylation motifs and RM-systems in the genome of *C. coli* BfR-CA-9557

In addition to reconstruction of the genome sequence SMRT sequencing allows determination of base modification by analysis of the sequencing kinetics. Using the SMRT Analysis Modification and Motif detection, we could identify 45,881 putatively *N*-6-methylated adenines (^m6^A, ~2.7 % of genome), 1788 4-methylated cytosines (^m4^C, ~0.1 %) and further 53,350 rather unspecific “modified bases” where the type of modification was not recognized by the software (Additional file 5: Figure S3).

**Fig. 3:**
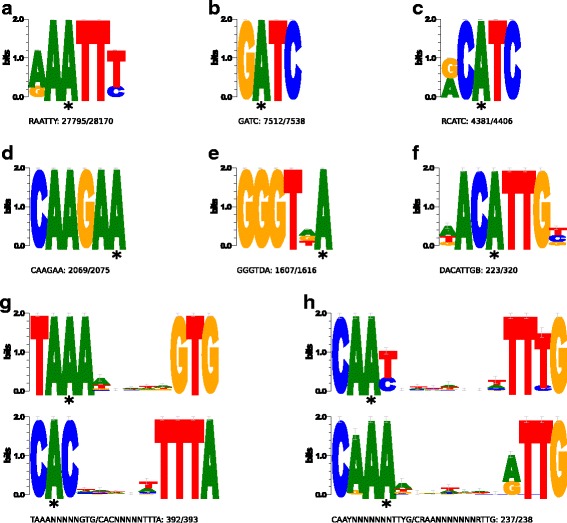
Sequence logos of eight methylation motifs. The consensus sequences of all eight motifs are depicted as sequence logo as obtained by the WebLogo 3 server (weblogo.threeplusone.com/create.cgi). The height of each stack indicates the degree of conservation (bits). The height of the letters represents the relative frequency of the base. The asterisk under a particular letter indicates the modified/methylated base. The two motifs in G and in H are partner motifs that are methylated at both strands. All motifs are recognized by *N*-6 adenine-specific methyltransferases

Methylated bases were arranged within eight different dominant methylation motifs (Table 3, Fig. 3). All motifs are recognized by *N*-6 adenine-specific methyltransferases. A small fraction (2.8 %) of ^m6^A bases were not clustered into any of the motifs, and no consensus motif could be identified for either ^m4^C-methylated bases or the majority of other unspecific modified bases.

**Table 3:** Methylation motifs of *C. coli* BFR-CA-9557

No.	Motif	Modified Position	Modification Type	% Motifs Detected	# of Motifs Detected	# of Motifs in Genome	Mean Modi-fication QV^1^	Mean Motif Coverage	Partner Motif
A	RAATTY	3	^m6^A	98.67	27795	28170	309.40	235.73	RAATTY
B	GATC	2	^m6^A	99.66	7512	7538	344.25	257.15	GATC
C	RCATC	3	^m6^A	99.43	4381	4406	274.48	256.54	
D	CAAGAA	6	^m6^A	99.71	2069	2075	290.35	249.45	
E	GGGTDA	6	^m6^A	99.44	1607	1616	327.70	254.15	
F	DACATTGB	4	^m6^A	69.69	223	320	80.60	255.05	
G1	TAAANNNNNGTG	3	^m6^A	99.75	392	393	271.96	259.91	CACNNNNNTTTA
G2	CACNNNNNTTTA	2	^m6^A	99.75	392	393	331.81	263.02	TAAANNNNNGTG
H1	CAAYNNNNNNNTTYG	3	^m6^A	99.58	237	238	316.89	246.49	CRAANNNNNNNRTTG
H2	CRAANNNNNNNRTTG	4	^m6^A	99.58	237	238	276.97	247.15	CAAYNNNNNNNTTYG

^1^ QV = quality value

Methylation motifs were identified using the PacBio SMRT Analysis software (see Methods). Column 2 shows the sequence consensus of the motif, whereby non-uniform positions are represented by IUPAC ambiguity codes. Column 3 and 4 denote the position of the modified base within the motif and the type of methylation. Column 5 represents the fraction of a motif’s occurrences in the genome (column 7) for which a methylation has been detected (column 6). Column 8 and 9 denote the average modification quality (in Phred Q-scores) and average coverage of motifs detected as modified. The last column shows the partner motif, i.e. the reverse complement of the motif

The motifs G1 & G2 and H1 & H2 (Table 3) are partner motifs and motifs A and B represent palindromic sequences, all of these partners containing methylated bases on both strands. In contrast, bases of motifs C, D, E, and F were methylated on only one strand. The major methylation motifs A, B, C, E, G, and H were almost completely methylated across their occurrences within the genome. The percentage of methylation ranges from 98.67 % to 99.75 %. In contrast, only 69.69 % of the ^m6^A methylation motif F sequences were methylated.

Using the recognition sequence search feature within the Restriction Enzyme Database – REBASE [56], only two out of the eight motifs (GATC, RAATTY) could be matched to existing recognition sequences of restriction systems (both Type II; http://rebase.neb.com/cgi-bin/pacbioget?17032). The remaining motifs represent yet unknown recognition sequences with the longer motifs (G and H) most likely being specific to *C. coli* or to this particular strain.

Using the REBASE sequence search feature and the NCBI, G2L and Prodigal/Prokka annotations four ORFs encoding subunits of Type I, seven ORFs encoding subunits of Type II, and three ORFs encoding subunits of Type IV RM-systems have been identified (Table 4). Only two of the REBASE predicted recognition sequences correspond to a specific motif detected by SMRT sequencing, namely: GAATTC/RAATTY (motif A) recognized by the DNA modification methylase (Adenine-specific methyltransferase) FokIM_2 (ORF #02605) and GATC (motif B) recognized by the DNA modification methyltransferase DpnA (ORF #2895). Additionally REBASE predicts a second candidate for a GATC-specific DNA modification methyltransferase in ORF #8910. In contrast, RAST and Prodigal predict a 16S rRNA (guanine(966)-N(2))-methyltransferase for this ORF.

**Table 4:** Putative *C. coli* BFR-CA-9557 restriction modification systems

ORF #^1^	Strand	Position in genome	Description	Type/subunit	Predicted rec. seq.
465	+	86828-89146	*hsdR*, Type I restriction-modification system2C restriction subunit R (EC 3.1.21.3)	I/R	-
485	+	92205-93437	*hsdS*, Type I restriction-modification system2C specificity subunit S (EC 3.1.21.3), EcoKI specificity protein	I/S	-
495	+	94709-96196	Type I restriction-modification system2C DNA-methyltransferase subunit M (EC 2.1.1.72)	I/M	-
6540	-	1246404-1250480	Type I restriction-modification system2C DNA-methyltransferase subunit M (EC 2.1.1.72) / Type I restriction-modification system2C specificity subunit S (EC 3.1.21.3)	I/MS	(GAGNNNNNGT)^3,4^
720	+	139123-140043	fokIM_1, Modification methylase FokI, (EC 2.1.1.72), homologue to ulcer associated adenine specific DNA methyltransferase	II/M	(GGATG)^3^
1470	-	275768-271959	Type IIS restriction enzyme Eco57I	II/S	-
2605	+	488272-489375	DNA modification methylase (Adenine-specific methyltransferase), fokIM_2, Modification methylase FokI, (EC 2.1.1.72)	II/M	GAATTC
2895	+	541751-542602	DNA modification methyltransferase, *dpnA*, Modification methylase DpnIIB (EC 2.1.1.72)	II/M	GATC
6520	-	1240865-1243951	*N*-6 adenine specific DNA methyltransferase	II/M	-
7730	+	1464470-1465285	DNA adenine methylase, dpnM, Modification methylase DpnIIA, EC 2.1.1.72	II/M	-
8910	+	1701685-1702374	16S rRNA (guanine(966)-N(2))-methyltransferase (EC 2.1.1.171)	II/M	GATC^5^
2225	+	409683-409970	McrBC 5-methylcytosine-specific restriction endonuclease system2C McrB subunit2C putative	IV/R	-
2230^2^	+	409982-411148	McrBC 5-methylcytosine-specific restriction endonuclease system2C McrB_1 subunit2C putative	IV/R	-
2230^2^	+	411208-412158	McrBC 5-methylcytosine-specific restriction endonuclease system2C McrB_2 subunit2C putative	IV/R	-
2235	+	412124-413494	McrBC 5-methylcytosine-specific restriction enzyme subunit McrC	IV/R	-

^1^ORF # according to the NCBI annotation pipeline for RAST and Prodigal ORF # see Additional files 3 and 4

^2^disrupted ORF

^3^predicted sequence does not correspond to any motif detected by SMRT sequencing

^4^According to REBASE ORF #6540 encodes a Type II RM-system

^5^In contrast to RAST and Prodigal REBASE predicts a second candidate for a GATC-specific DNA modification methyltransferase for this ORF

Restriction modification systems have been identified as outlined in section “Methods”. Column 1 to 3 denote the number, strand direction and genome position of the ORF as identified by the NCBI annotation pipeline. Column 4 contains the description of the ORF in terms of aggregated annotations from NCBI, RAST and Prodigal. The type and predicted recognition sequence of the motif are shown in columns 5 and 6

The two recognition sequences GAGNNNNNGT and GGATG predicted for the Type I restriction-modification system2C DNA-methyltransferase subunit M (ORF #6540) and modification methylase FokI homologue (ORF #720), respectively, do not correspond to any motif detected by SMRT sequencing in the genome of *C. coli* BfR-CA-9557.

REBASE search hits to the remaining ten RM-system subunit genes have not been associated with a specific recognition sequence. Therewith, REBASE was not able to predict a corresponding RM-system subunit for the motifs C, D, E, F, G, and H. This is most likely due to the fact that besides motifs A (RAATTY) and B (GATC) none of the published *C. jejuni* and *H. pylori* methylomes contains one of these motifs [49, 50, 57].

Motif B (GATC) is present in the methylomes of *H. pylori* 26695 and J99-R3 [57] but not in any of the publicly available *C. jejuni* methylomes. In the two *H. pylori* genomes ORFs *hp0092* and *jhp0085* encoding a RM-system IIM subunit have been assigned to this motif [58, 59]. The *C. coli* BfR-CA-9557 homologue of *hp0092* (*H. pylori* 26695) demonstrates a sequence identity of 76 % (553/730; Query coverage: 86 %).

In contrast, the RAATTY motif (A) has not been detected in one of the two *H. pylori* methylomes, but is was present in *C. jejuni* F38011, NCTC 11168 as well as 81–176 and in all three isolates a corresponding RM-system subunit has been assigned [50, 57]. The *C. coli* BfR-CA-9557 homologue of *cj0208* (NCTC 11168) demonstrates a sequence identity of 85 % (893/1050; Query coverage: 95 %).

The biological implications of this particular subset of RM-systems are difficult to predict, especially since the majority of methylation motifs and methylases in the genome of *C. coli* BFR-CA-9557 are quite novel. From the closely related bacterial species *H. pylori* it has been described in detail that RM-systems play a crucial role in forming strong barriers to prevent uptake of both plasmid and chromosomal DNA [60]. *H. pylori* bacterial cells are inherently very competent for DNA uptake, but this competence for DNA uptake varies significantly between specific strains. It has been observed that strains showing a very low endogenous RM-system activity demonstrate the highest transformation efficiency. Especially the presence of an RM-system homologous to *Mbo*I, which has been used for isoschizomer digestion assays in our study, has been described as major component of the *H. pylori* transformation barrier [60]. This *Mbo*I homologous RM-system named *Hpy*III is highly conserved among *H. pylori* strains and mediates protection against *Mbo*I digestion.

In *Mbo*I sensitive strains the *hpyIIIR* gene was found absent but a homologue to *C. jejuni cj1602*, namely *hrgA*, was detected. *HpyIIIR* negative but *hrgA* positive *H. pylori* strains have been associated with gastric cancer in Asian patients, while the pathogenic mechanism remains unclear [61].

Besides this role for DNA uptake the high intra-species variety in RM-system configuration and therewith the highly diverse methylation status of *H. pylori* chromosomal DNA was suggested to serve as a new typing system [62]. This DNA methylation based typing system may help to discriminate *H. pylori* isolates and as well isolates of related bacterial species like *C. jejuni* or *C. coli* for epidemiological and clinical purposes.

## Conclusions

The objective of the study was to analyze the methylome of a clade 1 *C. coli* isolate using SMRT sequencing. The *C. coli* isolate BfR-9557 was tested positive in 5’-G^m^ATC-3’ specific isoschizomer digestion assays and hence chosen for SMRT sequencing. Using one SMRT cell the complete genome of *C. coli* BfR-CA-9557 was sequenced and assembled into a single contig of 1.7 Mbp. The genome contains a CJIE1-like element prophage. SMRT Analysis Modification and Motif detection have identified eleven different dominant methylation motifs, while 14 RM-system subunits have been identified using REBASE and three different annotation pipelines. Only two of the REBASE predicted recognition sequences correspond to a specific motif detected by SMRT sequencing. The remaining 9 motifs did not correspond to any database record and are therefore characteristic for *C. coli* or at least for *C. coli* BfR-CA-9557. However, no comparable motifs have been described in the related microbial species *C. jejuni* or *H. pylori*.

## Methods

### Bacterial isolates, culture conditions, DNA extraction and MLST typing

50 *C. coli* isolates of different origin were obtained from the Federal Institute for Risk Assessment (BfR) in Berlin, Germany. The bacterial isolates were cultured on Columbia agar base (Merck) supplemented with 5 % sheep blood (BA) and incubated at 42 °C under microaerophilic conditions (5 % O_2_, 10 % CO_2_, 85 % N_2_) for 18 hours prior to genomic DNA extraction. Genomic DNA of all *C. coli* isolates was extracted using the QIAamp DNA Mini Kit (Qiagen) according to the manufacturer’s instructions.

Species confirmation was performed using MALDI Biotyper system (Bruker Daltonics, Bremen, Germany). Results with MALDI Biotyper identification score values ≥2.000 were considered correct. Additionally multiplex PCR was used to discriminate between *C. jejuni* and *C. coli* [63, 64].

The MLS-type was established using amplification and sequencing primers reported before [65]. The cycling conditions were 94 °C for 1 min, followed by 35 cycles of 94 °C for 120 s, 50 °C for 60 s, 72 °C for 60 s, followed by a final elongation step of 72 °C for 5 min [65]. Amplicons of the seven genes included in the *C. jejuni*/*C. coli* MLST scheme were sent for sequencing to Seqlab Sequence Laboratories GmbH (Göttingen, Germany) using 10 pmol of the respective sequencing primer.

### *5’-G*^*m*^*ATC-3’ specific* Isoschizomer digestion assay

Screening for a *C. coli* strain that methylates adenines in G^(m)^ATC sequences was performed using isoschizomer digestion assays in order to identify a *C. coli* strain expressing a Dam homologue. Genomic/chromosomal bacterial DNA was digested by the three restriction enzymes *Mbo*I, *Dpn*I and *Sau*3AI. *Mbo*I is responsive to Dam methylation. Therefore adenosyl-methylated GATC sequences become refractory to cleavage by *Mbo*I but turn susceptible to *Dpn*I cleavage. Additionally the restriction endonuclease *Sau*3AI that is insensitive to adenosyl-methylation by the Dam system was used as digestion control [51].

### Antimicrobial susceptibility testing

*C. jejuni* BfR-CA-9557 antimicrobial susceptibility to quinolones (ciprofloxacin) macrolides (erythromycin), aminoglycosides (gentamicin, streptomycin) and tetracyclines (tetracycline) was tested using the disc diffusion method according to the standards of the European Committee on Antimicrobial Susceptibility Testing (ESCMID) [66] and the microdilution assay according to the standards of the Clinical and Laboratory Standards Insitute (CLSI) [67], using cut-off values set by EUCAST (www.eucast.org). Antimicrobial test discs (ciprofloxacin, erythromycin, gentamicin, tetracycline) and EUCAMP2 microtiter plates were obtained from Oxoid/ThermoFisher Scientific (Wesel, Germany). For MIC analysis the Sensititre semiautomated system (Trek Diagnostic Systems, Inc, Cleveland, Ohio) was used.

### Library Preparation and Sequencing

Genomic DNA of *C. coli* BfR-CA-9557 (DSM 100395) was extracted using the QIAamp DNA Mini Kit (Qiagen) according to the manufacturer’s instructions. The DNA libraries have been prepared following the PacBio guidelines and sequenced on a SMRT cell using Pacific Biosciences RS sequencing technology (Pacific Biosciences, Menlo Park, USA) at Functional Genomics Center Zürich (FGCZ, Switzerland). Input genomic DNA concentration was measured using a Qubit Fluorometer dsDNA Broad Range assay (Life Technologies, Carlsbad, USA; p/n 32850). The SMRT bell was produced using the DNA Template Prep Kit 1.0 (Pacific Biosciences; p/n 100-259-100). 10 μg of gDNA were mechanically sheared to an avarage size distribution of 10Kb, using a Covaris gTube (Kbiosciences, Hoddesdon, UK; p/n 520079). A Bioanalyzer 2100 12 K DNA Chip assay (Agilent Technologies, Santa Clara, USA; p/n 5067–1508) was used to assess the fragment size distribution. 5 μg of sheared gDNA were DNA damage repaired and end-repaired using polishing enzymes. A blunt end ligation reaction followed by exonuclease treatment was performed to create the SMRT bell template. A Blue Pippin device (Sage Science, Beverly, USA) was used to size select the SMRT bell template and enrich the big fragments > 8Kbp. The sized selected library was quality inspected and quantified on the Agilent Bioanalyzer 12Kb DNA Chip and on a Qubit Fluorimeter.

A ready-to-sequence SMRT bell-polymerase Complex was created using the P6 DNA/Polymerase binding kit 2.0 (Pacific Biosciences, Menlo Park, USA; p/n 100-236-500) according to the manufacturer instructions.

The Pacific Biosciences RS2 instrument was programmed to load and sequence the sample on a single SMRT cell v3.0 (Pacific Biosciences p/n100-171-800), taking one movie of 120 minutes.

The MagBead loading method (PacBio, Menlo Park, USA; p/n 100-133-600) was chosen in order to improve the enrichment the longer fragments.

At the end of the run, a sequencing report was generated for every cell, via the SMRT portal. Thereby, the adapter dimer contamination, the sample loading efficiency, the obtained average read-length and the number of filtered sub-reads have been assessed.

### Sequence analysis

Processing of the raw SMRT cell data was performed using the Pacific Biosciences SMRT Analysis System (version 2.3, January 2015; PacBio, Menlo Park, USA).

For de novo assembly of the *C. coli* BFR-CA-9557 genome the high-quality Hierarchical Genome Assembly Process (RS_HGAP_Assembly.2) was used with standard parameters (for details on algorithms please read https://github.com/PacificBiosciences/Bioinformatics-Training/wiki/HGAP-in-SMRT-Analysis).

The assembled genome was annotated using the rapid annotation using subsystem technology platform (RAST, http://rast.nmpdr.org) [68–70] and the Prodigal/Prokka annotation pipeline [71] implemented at Göttingen Genome Laboratory (G2L).

For identification of methylated bases and modification motifs the RS_Modification_and_Motif_Analysis.1 protocol within the SMRT Analysis System was used with standard parameters on the basis of the previously assembled genome.

Putative restriction modification systems have been identified using the Restriction-ModificationFinder-1.0 server (https://cge.cbs.dtu.dk/services/Restriction-ModificationFinder-1.0/) based on the Restriction Enzyme database (REBASE, http://rebase.neb.com/rebase/rebase.html) [56].

Additionally, homologues of published *Campylobacter* and *Helicobacter* restriction modification [46–50] systems have been identified by BLAST search.

Additional checking for clustered regularly interspaced short palindromic repeats (CRISPRs) and CRISPR-associated (cas) genes was performed using CRISPR-finder (http://crispr.u-psud.fr/Server/Advanced.CRISPRfinder.php) [72].

### Ethics statement

Ethical clearance for the analysis was obtained from Ethics Committee of the University Medical Center Göttingen, Germany. As the bacterial isolates from human donors were already part of an anonymized strain collection and no evaluation including personal patient data has been performed the Ethics Committee of the University Medical Center Göttingen waived the need for written informed consent from the donor or the next of kin.

### Availability of supporting data

The genome is available at NCBI as *Campylobacter coli* BFR-CA-9557 with AB430 locus tags (BioProject Accession: PRJNA285481 ID: 285481; BioSample: SAMN03754337; GenBank ID: CP011777). Additionally RM-system and methylation motifs can be accessed via the index of the REBASE database (http://tools.neb.com/genomes/) or directly via this link: http://tools.neb.com/genomes/view.php?view_id=35944.

The bacterial isolate *Campylobacter coli* BFR-CA-9557 was deposited in the strain collection of the Leibniz-Institut DSMZ-Deutsche Sammlung von Mikroorganismen und Zellkulturen GmbH, Braunschweig, Germany (German Collection of Microorganisms and cell cultures); isolate ID: DSM 100395.

## Additional files

Additional file 1:
**Figure S1.** SMRT sequencing of Campylobacter coli BFR-CA-9557. (A) Read length distribution of 74,742 continuous long reads (CLR) obtained from a single SMRT cell after filtering for low quality. The black line depicts the cumulated amount of bases covered by reads of a minimum size as shown on the x-axis. (B) Distribution of read quality values (1 = 100 %) for 74,742 CLRs after filtering. The black line denotes the average length of reads with a quality at least as good as indicated on the x-axis. (C) Subread length distribution of 142,135 subreads (i.e. individual fragments of CLRs). (DOC 74 kb) Additional file 2:
**Figure S2.** Reference Coverage. (A) Reference coverage (number of read base pairs per position) of the polished assembly across the contig comprising 1,720,506 bp. Average reference coverage is 500.8-fold. (B) Histogram of reference coverage across the assembled contig. (DOC 73 kb) Additional file 3:
**RAST annotation of the** ***C. coli*** **BFR-CA-9557.** Annotation of the *C. coli* BFR-CA-9557 genome using the rapid annotation using subsystem technology platform (RAST, http://rast.nmpdr.org). (XLS 3605 kb) Additional file 4:
**Prodigal annotation of the** ***C. coli*** **BFR-CA-9557.** Annotation of the *C. coli* BFR-CA-9557 genome using the Prodigal/Prokka annotation pipeline. (XLS 727 kb) Additional file 5
**Figure S3.** Methylated bases in *C. coli* BFR-CA-9557. (A) Scatter plot of modification quality values and per-strand coverage of 101,019 bases detected as methylated in the *C. coli* BFR-CA-9557 genome. (B) Histogram of modification quality values for all bases. (DOC 115 kb)

## Abbreviations

CC: Clonal complex
CJIE1: *Campylobacter jejuni* integrated element 1
MLST: Multi-locus sequence typing
RM-system: Restriction modification system
ST: Sequence type
