# Supplementary figures and images for: SMRT sequencing of the Campylobacter coli BfR-CA-9557 genome sequence reveals unique methylation motifs
Source: BMC Genomics. 2015 Dec 21;16:1088. doi: 10.1186/s12864-015-2317-3 (PMC4687069; doi:10.1186/s12864-015-2317-3)

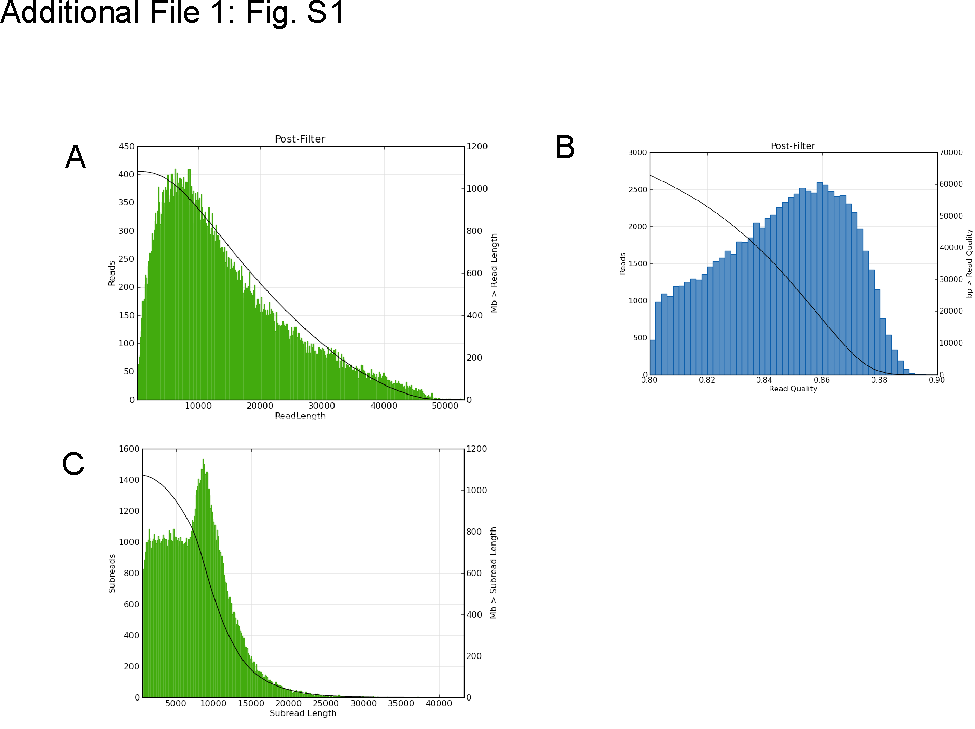

Supplement: Additional file 1: — Figure S1. SMRT sequencing of Campylobacter coli BFR-CA-9557. (A) Read length distribution of 74,742 continuous long reads (CLR) obtained from a single SMRT cell after filtering for low quality. The black line depicts the cumulated amount of bases covered by reads of a minimum size as shown on the x-axis. (B) Distribution of read quality values (1 = 100 %) for 74,742 CLRs after filtering. The black line denotes the average length of reads with a quality at least as good as indicated on the x-axis. (C) Subread length distribution of 142,135 subreads (i.e. individual fragments of CLRs). (DOC 74 kb) [file 12864_2015_2317_MOESM1_ESM.doc]

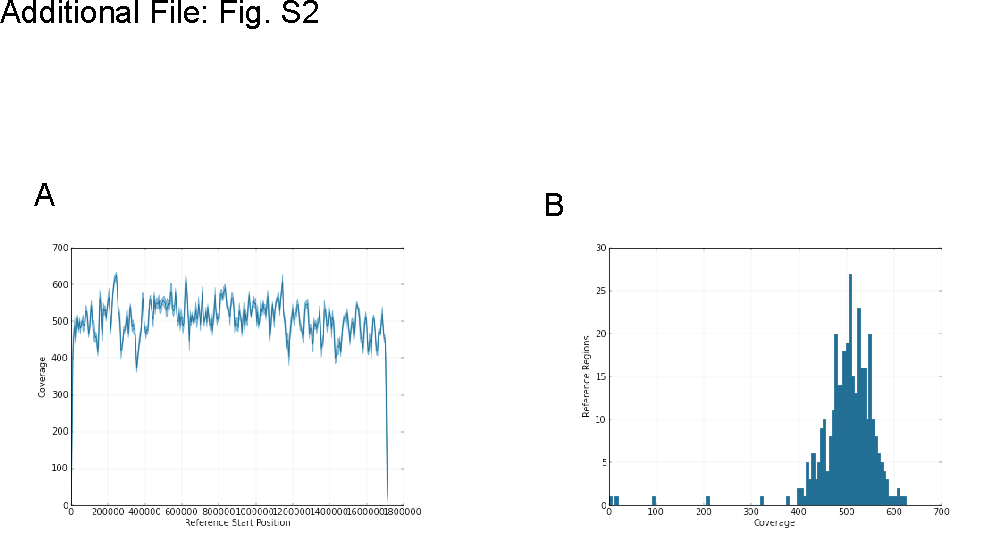

Supplement: Additional file 2: — Figure S2. Reference Coverage. (A) Reference coverage (number of read base pairs per position) of the polished assembly across the contig comprising 1,720,506 bp. Average reference coverage is 500.8-fold. (B) Histogram of reference coverage across the assembled contig. (DOC 73 kb) [file 12864_2015_2317_MOESM2_ESM.doc]

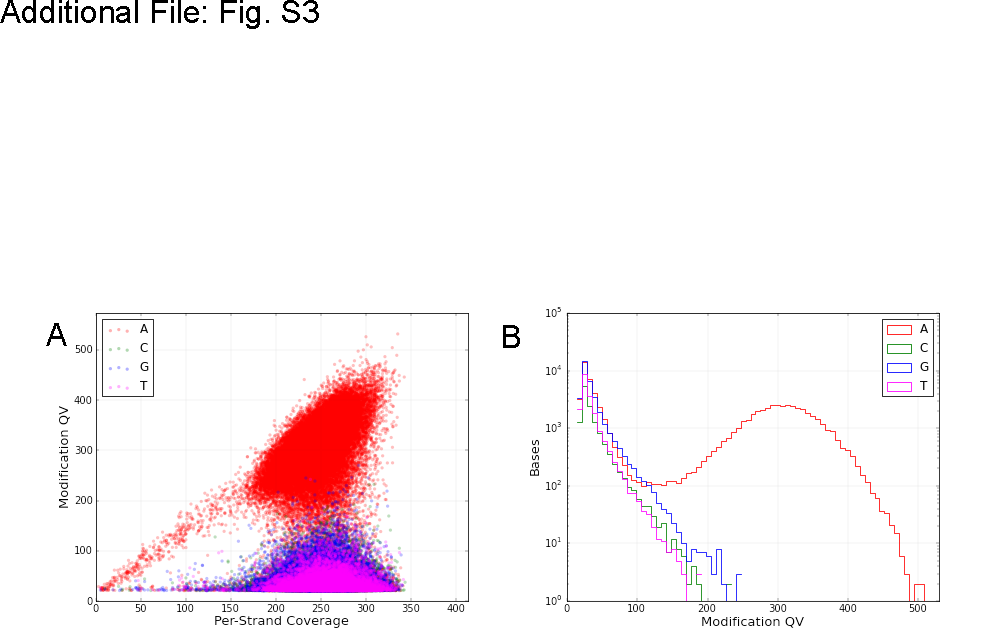

Supplement: Additional file 5 — Figure S3. Methylated bases in C. coli BFR-CA-9557. (A) Scatter plot of modification quality values and per-strand coverage of 101,019 bases detected as methylated in the C. coli BFR-CA-9557 genome. (B) Histogram of modification quality values for all bases. (DOC 115 kb) [file 12864_2015_2317_MOESM5_ESM.doc]
